# Quantum Hair on Colliding Black Holes

**DOI:** 10.3390/e22030301

**Published:** 2020-03-05

**Authors:** Lawrence Crowell, Christian Corda

**Affiliations:** 1 AIAS, Budapest 1011, Hungary; goldenfieldquaternions@gmail.com; 2 Department of Physics, Faculty of Science, Istanbul University, Istanbul 34134, Turkey; 3 International Institute for Applicable Mathematics and Information Sciences, B.M., Birla Science Centre, Adarshnagar, Hyderabad 500063, India

**Keywords:** colliding black holes, quantum hair, bohr-likr black holes

## Abstract

Black hole (BH) collisions produce gravitational radiation which is generally thought, in a quantum limit, to be gravitons. The stretched horizon of a black hole contains quantum information, or a form of quantum hair, which is a coalescence of black holes participating in the generation of gravitons. This may be facilitated with a Bohr-like approach to black hole (BH) quantum physics with quasi-normal mode (QNM) approach to BH quantum mechanics. Quantum gravity and quantum hair on event horizons is excited to higher energy in BH coalescence. The near horizon condition for two BHs right before collision is a deformed AdS spacetime. These excited states of BH quantum hair then relax with the production of gravitons. This is then argued to define RT entropy given by quantum hair on the horizons. These qubits of information from a BH coalescence should then appear in gravitational wave (GW) data.

## 1. Introduction

Quantum gravitation suffers primarily from an experimental problem. It is common to read critiques that it has gone off into mathematical fantasies, but the real problem is the scale at which such putative physics holds. It is not hard to see that an accelerator with current technology would be a ring encompassing the Milky Way galaxy. Even if we were to use laser physics to accelerate particles the energy of the fields proportional to the frequency could potentially reduce this by a factor of about $10^{6}$ so a Planck mass accelerator would be far smaller; it would encompass the solar system including the Oort cloud out to at least 1 light years. It is also easy to see that a proton-proton collision that produces a quantum black hole (BH) of a few Planck masses would decay into around a mole of daughter particles. The detection and track finding work would be daunting. Such experiments are from a practical perspective nearly impossible. This is independent of whether one is working with string theory or loop variables and related models. It is then best to let nature do the heavy lifting for us. Gravitation is a field with a coupling that scales with the square of mass-energy. Gravitation is only a strong field when lots of mass-energy is concentrated in a small region, such as a BH. The area of the horizon is a measure of maximum entropy any quantity of mass-energy may possess [1], and the change in horizon area with lower and upper bounds in BH thermodynamic a range for gravitational wave production. Gravitational waves produced in BH coalescence contains information concerning the BHs configuration, which is argued here to include quantum hair on the horizons. Quantum hair means the state of a black hole from a single microstate in no-hair theorems. Strominger and Vafa [2] advanced the existence of quantum hair using theory of D-branes and STU string duality. This information appears as gravitational memory, which is found when test masses are not restored to their initial configuration [3]. This information may be used to find data on quantum gravitation. There are three main systems in physics, quantum mechanics (QM), statistical mechanics and general relativity (GR) along with gauge theory. These three systems connect with each other in certain ways. There is quantum statistical mechanics in the theory of phase transitions, BH thermodynamics connects GR with statistical mechanics, and Hawking-Unruh radiation connects QM to GR as well. These are connections but are incomplete and there has yet to be any general unification or reduction of degrees of freedom. Unification of QM with GR appeared to work well with holography, but now faces an obstruction called the firewall [4]. Hawking proposed that black holes may lose mass through quantum tunneling [5]. Hawking radiation is often thought of as positive and negative energy entangled states where positive energy escapes and negative energy enters the BH. The state which enters the BH effectively removes mass from the same BH and increases the entanglement entropy of the BH through its entanglement with the escaping state. This continues but this entanglement entropy is limited by the Bekenstein bound. In addition, later emitted bosons are entangled with both the black hole and previously emitted bosons. This means a bipartite entanglement is transformed into a tripartite entangled state. This is not a unitary process. This will occur once the BH is at about half its mass at the Page time [6], and it appears the unitary principle (UP) is violated. In order to avoid a violation of UP the equivalence principle (EP) is assumed to be violated with the imposition of a firewall. The unification of QM and GR is still not complete. An elementary approach to unitarity of black holes prior to the Page time is with a Bohr-like approach to BH quantum physics [7,8,9], which will be discussed in next section. Quantum gravity hair on BHs may be revealed in the collision of two BHs. This quantum gravity hair on horizons will present itself as gravitational memory in a GW. This is presented according to the near horizon condition on Reissnor-Nordstrom BHs, which is $AdS_{2}\times S^{2}$, which leads to conformal structures and complementarity principle between GR and QM.

## 2. Bohr-Like Approach to Black Hole Quantum Physics

At the present time, there is a large agreement, among researchers in quantum gravity, that BHs should be highly excited states representing the fundamental bricks of the yet unknown theory of quantum gravitation [7,8,9]. This is parallel to quantum mechanics of atoms. In the 1920s the founding fathers of quantum mechanics considered atoms as being the fundamental bricks of their new theory. The analogy permits one to argue that BHs could have a discrete energy spectrum [7,8,9]. In fact, by assuming the BH should be the nucleus the “gravitational atom”, then, a quite natural question is—What are the “electrons”? In a recent approach, which involves various papers (see References [7,8,9] and references within), this important question obtained an intriguing answer. The BH quasi-normal modes (QNMs) (i.e., the horizon’s oscillations in a semi-classical approach) triggered by captures of external particles and by emissions of Hawking quanta, represent the “electrons” of the BH which is seen as being a gravitational hydrogen atom [7,8,9]. In References [7,8,9] it has been indeed shown that, in the the semi-classical approximation, which means for large values of the BH principal quantum number *n*, the evaporating Schwarzschild BH can be considered as the gravitational analogous of the historical, semi-classical hydrogen atom, introduced by Niels Bohr in 1913 [10,11]. Thus, BH QNMs are interpreted as the BH electron-like states, which can jump from a quantum level to another one. One can also identify the energy shells of this gravitational hydrogen atom as the absolute values of the quasi-normal frequencies [7,8,9]. Within the semi-classical approximation of this Bohr-like approach, unitarity holds in BH evaporation. This is because the time evolution of the Bohr-like BH is governed by a time-dependent Schrodinger equation [8,9]. In addition, subsequent emissions of Hawking quanta [5] are entangled with the QNMs (the BH electron states) [8,9]. Various results of BH quantum physics are consistent with the results of [8,9], starting from the famous result of Bekenstein on the area quantization [12]. Recently, this Bohr-like approach to BH quantum physics has been also generalized to the Large AdS BHs, see Reference [13]. For the sake of simplicity, in this Section we will use Planck units ($G=c=k_{B}=\hbar =\frac{1}{4\pi \epsilon_{0}}=1$). Assuming that *M* is the initial BH mass and that $E_{n}$ is the total energy emitted by the BH when the same BH is excited at the level *n* in units of Planck mass (then $M_{p}\;=\;1$), one gets that a discrete amount of energy is radiated by the BH in a quantum jump in terms of energy difference between two quantum levels [7,8,9]

(1) $$\begin{array}{c} \Delta E_{n_{1}\to n_{2}}\equiv E_{n_{2}}-E_{n_{1}}=M_{n_{1}}-M_{n_{2}}\\ =\sqrt{M^{2}-\frac{n_{1}}{2}}-\sqrt{M^{2}-\frac{n_{2}}{2}}, \end{array}$$

This equation governs the energy transition between two generic, allowed levels $n_{1}$ and $n_{2}>n_{1}$ and consists in the emission of a particle with a frequency $\Delta E_{n_{1}\to n_{2}}$ [7,8,9]. The quantity $M_{n}$ in Equation (1), represents the residual mass of the BH which is now excited at the level n. It is exactly the original BH mass minus the total energy emitted when the BH is excited at the level *n* [8,9]. Then, $M_{n}=M-E_{n},$ and one sees that the energy transition between the two generic allowed levels depends only on the two different values of the BH principal quantum number and on the initial BH mass [7,8,9]. An analogous equation works also in the case of an absorption, See References [7,8,9] for details. In the analysis of Bohr [10,11], electrons can only lose and gain energy during quantum jumps among various allowed energy shells. In each jump, the hydrogen atom can absorb or emit radiation and the energy difference between the two involved quantum levels is given by the Planck relation (in standard units) E=hν. In the BH case, the BH QNMs can gain or lose energy by quantum jumps from one allowed energy shell to another by absorbing or emitting radiation (Hawking quanta). The following intriguing remark finalizes the analogy between the current BH analysis and Bohr’s hydrogen atom. The interpretation of Equation (1) is the energy states of a particle, that is the electron of the gravitational atom, which is quantized on a circle of length [7,8,9]

(2) $$L=4\pi \left(M+\sqrt{M^{2}-\frac{n}{2}}\right).$$

Hence, one really finds the analogous of the electron traveling in circular orbits around the nucleus in Bohr’s hydrogen atom. One sees that it is also

(3) $$\begin{array}{c} M_{n}=\sqrt{M^{2}-\frac{n}{2}} \end{array}.$$

Thus the uncertainty in a clock measuring a time *t* becomes, with the Planck mass is equal to 1 in Planck units,

(4) $$\frac{\delta t}{t}=\frac{1}{2M_{n}}=\frac{1}{\sqrt{M^{2}-\frac{n}{2}}},$$

which means that the accuracy of the clock required to record physics at the horizon depends on the BH excited state, which corresponds to the number of Planck masses it has. More in general, from the Bohr-like approach to BH quantum physics it emerges that BHs seem to be well defined quantum mechanical systems, having ordered, discrete quantum spectra. This issue appears consistent with the unitarity of the underlying quantum gravity theory and with the idea that information should come out in BH evaporation, in agreement with a known result of Page [6]. For the sake of completeness and of correctness, we stress that the topic of this Section, that is, the Bohr-like treatment of BH quantum physics, is not new. A similar approach was used by Bekenstein in 1997 [14] and by Chandrasekhar in 1998 [15].

## 3. Near Horizon Spacetime and Collision of Black Holes

This paper proposes how the quantum basis of black holes may be detected in gravitational radiation. Signatures of quantum modes may exist in gravitational radiation. Gravitational memory or BMS symmetries are one way in which quantum hair associated with a black hole may be detected [16]. Conservation of quantum information suggests that quantum states on the horizon may be emitted or entangled with gravitational radiation and its quantum numbers and information. In what follows a toy model is presented where a black hole coalescence excites quantum hair on the stretched horizon in the events leading up to the merger of the two horizons. The model is the Poincare disk for spatial surface in time. To motivate this we look at the near horizon condition for a near extremal black hole. The Reissnor-Nordstrom (RN) metric is

$$ds^{2}\;=\;-\left(1\;-\;\frac{2m}{r}\;+\;\frac{Q^{2}}{r^{2}}\right)dt^{2}\;+\;{\left(1\;-\;\frac{2m}{r}\;+\;\frac{Q^{2}}{r^{2}}\right)}^{-1}dr^{2}\;+\;r^{2}d\Omega^{2}.$$

Here *Q* is an electric or Yang-Mills charge and *m* is the BH mass. In previous section, considering the Schwarzschild BH, we labeled the BH mass as *M* instead. The accelerated observer near the horizon has a constant radial distance. For the sake of completeness, we recall that the Bohr-like approach to BH quantum physics has been also partially developed for the Reissnor-Nordstrom black hole (RNBH) in Reference [14]. In that case, the expression of the energy levels of the RNBH is a bit more complicated than the expression of the energy levels of the Schwarzschild BH, being given by (in Planck units and for small values of *Q*) [14]

(5) $$E_{n}≃m-\sqrt{m^{2}+\frac{q^{2}}{2}-Qq-\frac{n}{2}},$$

where *q* is the total charge that has been loss by the BH excited at the level *n*. Now consider

$$\rho \;=\;\int_{r_{+}}^{r}dr\sqrt{g_{rr}}\;=\;\int_{r^{+}}^{r}\frac{dr}{\sqrt{1\;-\;2m/r\;+\;Q^{2}/r^{2}}}$$

with lower integration limit $r_{+}$ is some small distance from the horizon and the upper limit *r* removed from the black hole. The result is

$$\rho \;=\;m\;log\left[\sqrt{r^{2}\;-\;2mr\;+\;Q^{2}}\;+\;r\;-\;m\right]\;+\;\sqrt{r^{2}\;-\;2mr\;+\;Q^{2}}{|}_{r_{+}}^{r}$$

with a change of variables ρ=ρ(r) the metric is

(6) $$ds^{2}\;=\;{\left(\frac{\rho }{m}\right)}^{2}dt^{2}\;-\;{\left(\frac{m}{\rho }\right)}^{2}d\rho^{2}\;-\;m^{2}d\Omega^{2},$$

where on the horizon ρ→r. This is the metric for $AdS_{2}\times S^{2}$ for $AdS_{2}$ in the (t,ρ) variables tensored with a two-sphere $S^{2}$ of constant radius =m in the angular variables at every point of $AdS_{2}$. This metric was derived by Carroll, Johnson and Randall [17]. In Section 4 it is shown this hyperbolic dynamics for fields on the horizon of coalescing BHs is excited. This by the Einstein field equation will generate gravitational waves, or gravitons in some quantum limit not completely understood. This GW information produced by BH collisions will reach the outside world highly red shifted by the tortoise coordinate $r^{*}\;=\;r^{\prime }\;-\;r\;-\;2m\;ln\left|1\;-\;2m/r\right|$. For a 30 solar mass BH, which is mass of some of the BHs which produce gravitational waves detected by LIGO, the wavelength of this ripple, as measured from the horizon to δr∼λ

$$\delta r^{\prime }\;=\;\lambda \;-\;2m\;ln\left(\frac{\lambda }{2m}\right)\;≃\;2\times 10^{6}m.$$

A ripple in spacetime originating an atomic distance $10^{-10}$ m from the horizon gives a ν=150 Hz signal, detectable by LIGO [18]. Similarly, a ripple $10^{-13}$ to $10^{-17}$ cm from the horizon will give a $10^{-1}$ Hz signal detectable by the eLISA interferometer system [19]. Thus, quantum hair associated with QCD and electroweak interactions that produce GWs could be detected. More exact calculations are obviously required. Following Reference [20], one can use Hawking’s periodicity argument from the RN metric in order to obtain an “effective” RN metric which takes into account the BH dynamical geometry due to the subsequent emissions of Hawking quanta. Hawking radiation is generated by a tunneling of quantum hair to the exterior, or equivalently by the reduction in the number of quantum modes of the BH. This process should then be associated with the generation of a gravitational wave. This would be a more complete dynamical description of the response spacetime has to Hawking radiation, just as with what follows with the converse absorption of mass or black hole coalescence. This will be discussed in a subsequent paper. These weak gravitons produced by BH hair would manifest themselves in gravitational memory. The Bondi-Metzner-Sachs (BMS) symmetry of gravitational radiation results in the displacement of test masses [21]. This displacement requires an interferometer with free floating mirrors, such as what will be available with the eLISA system. The BMS symmetry is a record of YM charges or potentials on the horizon converted into gravitational information. The BMS metric provide phenomenology for YM gauge fields, entanglements of states on horizons and gravitational radiation. The physics is correspondence between YM gauge fields and gravitation. The BHs coalescence is a process which converts qubits on the BHs horizons into gravitons. Two BHs close to coalescence define a region between their horizons with a vacuum similar to that in a Casimir experiment. The two horizons have quantum hair that forms a type of holographic “charge” that performs work on spacetime as the region contracts. The quantum hair on the stretched horizon is raised into excited states. The ansatz is made that $AdS_{2}\times S^{2}$ for two nearly merged BHs is mapped into a deformed $AdS_{4}$ for a small region of space between two event horizons of nearly merged BHs. The deformation is because the conformal hyperbolic disk is mapped into a strip. In one dimension lower, the spatial region is a two dimensional hyperbolic strip mapped from a Poincare disk with the same SL(2,R) symmetry. The manifold with genus *g* for charges has Euler characteristic χ=2g−2 and with the 3 dimensions of SL(2,R) this is the index 6g−6 for Teichmuller space [21]. The SL(2,R) is the symmetry of the spatial region with local charges modeled as a U(1) field theory on an $AdS_{3}$. The Poincare disk is then transformed into $H_{p}^{2}$ that is a strip. The $H_{p}^{2}\;\subset \;AdS_{3}$ is simply a Poincare disk in complex variables then mapped into a strip with two boundaries that define the region between the two event horizons.

## 4. AdS Geometry in BH Coalescence

The near horizon condition for a near extremal black hole approximates $AdS_{2}\times S^{2}$. In Reference [17] the extremal blackhole replaces the spacelike region in $\left(r_{+},\;r_{-}\right)$ with $AdS_{2}\times S^{2}$. For two black holes in near coalescence there are two horizons, that geodesics terminate on. The region between the horizons is a form of Kasner spacetime with an anisotropy in dynamics between the radial direction and on a plane normal to the radial direction. In the appendix it is shown this is for a short time period approximately an $AdS_{4}$ spacetime. The spatial surface is a three-dimensional Poincare strip, or a three-dimensional region with hyperbolic arcs. This may be mapped into a hyperbolic space $H^{3}$. This is a further correlation between anti-de Sitter spacetimes and black holes, such as seen in AdS/BH correspondences [22]. The region between two event horizons is argued to be approximately $AdS_{4}$ by first considering the two BHs separated by some distance. There is an expansion of the area of the $S^{2}$ that is then employed with the $AdS_{2}\times S^{2}$. We then make some estimates on the near horizon condition for black holes very close to merging. To start consider the case of two equal mass black holes in a circular orbit around a central point. We consider the metric near the center of mass r=0 and the distance between the two black holes d>>2m. In doing this we may get suggestions om how to model the small region between two black holes about to coalesce. An approximate metric for two distant black holes is of the form

$$ds^{2}\;=\;\left(1\;-\;\frac{2m}{\left|r+d\right|}\;-\;\frac{2m}{\left|r+d\right|}\right)dt^{2}\;-\;{\left(1\;-\;\frac{2m}{\left|r+d\right|}\;-\;\frac{2m}{\left|r+d\right|}\right)}^{-1}dr^{2}\;-\;r^{2}\left(d\theta^{2}\;+\;sin^{2}\theta d\Phi^{2}\right),$$

where dΦ=dϕ+ωdt, for ω the angular velocity of the two black holes around r=0. With the approximation for a moderate Keplerian orbit we may then write this metric as This metric is approximated with the binomial expansion to $O(r^{2})$ and O(ω) as

$$ds^{2}\;=\;\left(1\;-\;\frac{2m}{d}\left(1\;+\;2\frac{r^{2}}{d^{2}}\right)\right)dt^{2}\;-\;{\left(1\;-\;\frac{2m}{d}\left(1\;+\;2\frac{r^{2}}{d^{2}}\right)\right)}^{-1}dr^{2}$$

$$-\;2r^{2}\omega \sin\theta d\phi dt\;-\;r^{2}\left(d\theta^{2}\;+\;sin^{2}\theta d\phi^{2}\right).$$

$g_{tt}$ is similar to the $AdS_{2}$$g_{tt}$ metric term plus constant terms and and similarly $g_{rr}$. It is important to note this approximate metric has expanded the measure of the angular portion of the metric. This means the 2-sphere with these angle measures has more “area” than before from the contribution of angular momentum.

The Ricci curvatures are

$$R_{tt}\;=\;R_{rr}\;≃\;-\frac{4m}{d},\;R_{t\phi }\;≃\;\left[4\left(1\;+\;\frac{4m}{d}\right)\;+\;\frac{16mr^{2}}{d^{3}}\right]\omega sin^{2}\theta ,$$

$$R_{\phi \phi }\;=\;g_{t\phi }g^{tt}R_{t\phi }\;≃\;-8r^{2}\omega^{2}sin^{4}\theta \;+\;O\left(\frac{\omega^{2}}{d}\right),\;R_{\theta \theta }\;=\;0,$$

where $O(d^{-2})$ terms and higher are dropped. The $R_{rr}$ and $R_{t\phi }$ Ricci curvature are negative and $R_{t\phi }$ positive. The 2-surface in r,ϕ coordinates has hyperbolic properties. This means we have at least the embedding of a deformed version of $AdS_{3}$ in this spacetime. This exercise expands the boundary of the disk $D^{2}$, in a 2-spacial subsurface, with boundary around each radial distance so there is an excess angle or “wedge” that gives hyperbolic geometry.

The (t,ϕ) curvature components comes from the Riemannian curvature $R_{r\phi tr}$$=\;-\frac{1}{2}\omega \alpha^{-1}$ and its contribution to the geodesic deviation equation along the radial direction is

$$\frac{d^{2}r}{ds^{2}}\;+\;R_{\phi tr}^{r}U^{t}U^{\phi }r\;=\;0$$

or that for $U^{t}\;≃\;1$ and $U^{\phi }\;≃\;\omega$

$$\frac{d^{2}r}{dt^{2}}\;≃\;\frac{1}{2}\omega^{2}r.$$

This has a hyperbolic solution $r\;=\;r_{0}cosh\left(\frac{1}{\sqrt{2}}\omega t\right)$. The $U^{\phi }$ will have higher order terms that may be computed in the dynamics for ϕ Similarly the geodesic deviation equation for ϕ is

$$\frac{d^{2}\phi }{ds^{2}}\;+\;R_{rtr}^{\phi }U^{t}U^{r}r\;=\;0$$

or cryptically

$$\frac{d^{2}\phi }{dt^{2}}\;≃\;\mathrm{Riem}\;A\;cosh\left(\alpha t\right)sinh\left(\alpha t\right),$$

for $\mathrm{Riem}\;\to \;R_{\mathrm{rtr}}^{Œ}$. This has an approximately linear form for small *t* that turns around into exponential or hyperbolic forms for larger time. The spatial manifold in the (r,ϕ) variables then have some hyperbolic structure.

It is worth a comment on the existence of Ricci curvatures for this spacetime. The Schwarzschild metric has no Ricci curvature as a vacuum solution. This 2-black hole solution however is not exactly integrable and so mass-energy is not localizable. This means there is an effective source of curvature due to the nonlocalizable nature of mass-energy for this metric. This argument is made in order to justify the ansatz the spacetime between two close event horizons prior to coalescence is $AdS_{4}$. Since most of the analysis of quantum field is in one dimension lower it is evident there is a subspace $AdS_{3}$. This is however followed up by looking at geometry just prior to coalescence where the $S^{2}$ has more area than it can bound in a volume. This leads to hyperbolic geometry. Above we argue there is an expansion of a disk boundary $\partial D^{2}$, and thus hyperbolic geometry. It is then assume this carries to one additional dimension as well. Now move to examine two black holes with their horizons very close. Consider a modification of the $AdS_{2}\times S^{2}$ metric with the inclusion of more “œarea” in the $S^{2}$ portion. The addition of area to $S^{2}$ is then included in the metric. In this fashion the influence of the second horizon is approximated by a change in the metric of $S^{2}$. The metric is then a modified form of the near horizon metric for a single black hole,

$$ds^{2}\;=\;{\left(\frac{r}{R}\right)}^{2}dt^{2}\;-\;{\left(\frac{R}{r}\right)}^{2}dr^{2}\;-\;\left(r^{2}\;+\;\rho^{2}\right)d\Omega^{2}.$$

The term ρ means there is additional area to the $S^{2}$ making it hyperbolic. The Riemann curvatures for this metric are:

$$R_{trtr}\;=\;-\frac{1}{r^{2}}\;-\;\frac{2\rho^{2}}{r^{2}\left(r^{2}\;+\;\rho^{2}\right)},\;R_{r\theta r\theta }\;=\;-\frac{\rho^{2}}{r^{2}\;+\;\rho^{2}},\;R_{r\phi r\phi }\;=\;-\frac{\rho^{2}}{r^{2}\;+\;\rho^{2}}sin^{2}\theta ,\;R_{\theta \phi \theta \phi }\;=\;\rho^{2}sin^{2}\theta$$

From these the Ricci curvatures are

$$R_{rr}\;=\;-\frac{1}{r^{2}}\;-\;2{\frac{\rho^{2}}{r^{2}\;+\;\rho^{2}}}^{2},\;R_{\theta \theta }\;=\;R_{\phi \phi }\;=\;-\left(1\;+\;\frac{R^{2}}{r^{2}}\right)\frac{\rho^{2}}{r^{2}\;+\;\rho^{2}}$$

are negative for small values of *r*. For r→0 all Ricci curvatures diverge Ric→−∞. The $R_{rr}$ diverges more rapidly, which gives this spacetime region some properties similar to a Kasner metric. However, $R_{rr}\;-\;R_{\theta \theta }$ is finite for r→∞. This metric then has properties of a deformed $AdS_{4}$. With the treatment of quantum fields between two close horizons before coalescence the hyperbolic space $H^{2}$ is considered as the spatial surface in a highly deformed $AdS_{3}$. A Poincare disk is mapped into a hyperbolic strip.

The remaining discussion will now center around the spatial hyperbolic spatial surface. In particular the spatial dimensions are reduced by one. This is then a BTZ-like analysis of the near horizon condition. The 2 dimensional spatial surface will exhibit hyperbolic dynamics for particle fields and this is then a model for the near horizon hair that occurs with the two black holes in this region.

For the sake of simplicity now reduce the dimensions and consider $AdS_{3}$ in 2 plus 1 spacetime. The near horizon condition for a near extremal black hole in 4 dimensions is considered for the BTZ black hole. This $AdS_{3}$ spacetime is then a foliations of hyperbolic spatial surfaces $H^{2}$ in time. These surfaces under conformal mapping are a Poincare disk. The motion of a particle on this disk are arcs that reach the conformal boundary as t→∞. This is then the spatial region we consider the dynamics of a quantum particle. This particle we start out treating as a Dirac particle, but the spinor field we then largely ignore by taking the square of the Dirac equation to get a Klein-Gordon wave. Define the *z* and $\bar{z}$ of the Poincare disk with the metric

$$ds_{p-disk}^{2}\;=\;R^{2}g_{z\bar{z}}dzd\bar{z}\;=\;R^{2}\frac{dzd\bar{z}}{1\;-\;z\bar{z}}$$

with constant negative Gaussian curvature $R\;=\;-4/R^{2}$. This metric $g_{z\bar{x}}\;=\;R^{2}/\left(1\;-\;\bar{z}z\right)$ is invariant under the SL(2,R)∼SU(1,1) group action, which, for g∈SU(1,1), takes the form

(7) $$z\;\to \;gz\;=\;\frac{az\;+\;b}{\bar{b}z\;+\;\bar{a}},\;g\;=\;\left(\begin{array}{cc} a & b\\ \bar{b} & \bar{a} \end{array}\right).$$

The Dirac equation $i\gamma^{\mu }D_{\mu }\psi \;+\;m\psi \;=\;0$, $D_{\mu }\;=\;\partial_{\mu }\;+\;iA_{\mu }$ on the Poincare disk has the Hamiltonian matrix

(8) $$H\;=\;\left(\begin{array}{cc} m & H_{w}\\ H_{w}^{*} & -m \end{array}\right)$$

for the Weyl Hamiltonians

$$H_{w}\;=\;\frac{1}{\sqrt{g_{z\bar{z}}}}\alpha_{z}\left(2D_{z}\;+\;\frac{1}{2}\partial_{z}\left(ln\;g_{z\bar{z}}\right)\right),$$

$$H_{w}^{*}\;=\;\frac{1}{\sqrt{g_{z\bar{z}}}}\alpha_{\bar{z}}\left(2D_{\bar{z}}\;+\;\frac{1}{2}\partial_{\bar{z}}\left(ln\;g_{z\bar{z}}\right)\right),$$

with $D_{z}\;=\;\partial_{z}\;+\;iA_{z}$ and $D_{\bar{z}}\;=\;\partial_{\bar{z}}\;+\;iA_{\bar{z}}$. here $\alpha_{z}$ and ${\bar{\alpha }}_{z}$ are the 2×2 Weyl matrices. Now consider gauge fields, in this case magnetic fields, in the disk. These magnetic fields are topological in the sense of the Dirac monopole with vanishing Ahranov-Bohm phase. The vector potential for this field is

$$A^{\phi }\;=\;-i\frac{\phi }{2}\left(\frac{dz}{z}\;-\;\frac{d\bar{z}}{\bar{z}}\right).$$

the magnetic field is evaluated as a line integral around the solenoid opening, which is zero, but the Stokes’ rule indicates this field will be $\phi \left(\bar{z}-z\right)/r^{2}$, for $r^{2}\;=\;\bar{z}z$. A constant magnetic field dependent upon the volume $V\;=\;\frac{1}{2}dz\wedge d\bar{z}$ in the space with constant Gaussian curvature $R\;=\;-4/R^{2}$

$$A^{v}\;=\;i\frac{BR^{2}}{4}\left(\frac{zd\bar{z}\;-\;\bar{z}dz}{1\;-\;\bar{z}z}\right).$$

The Weyl Hamiltonians are then

$$H_{w}\;=\;\frac{1\;-\;r^{2}}{R}e^{-i\theta }\left(\alpha_{z}\left(\partial_{r}\;-\;\frac{i}{r}\partial_{\theta }\;-\;\frac{\sqrt{\ell (\ell \;+\;1)}\;+\;\phi }{r}\;+\;i\frac{kr}{1\;-\;r^{2}}\right)\right)$$

(9) $$H_{w}^{*}\;=\;\frac{1\;-\;r^{2}}{R}e^{i\theta }\left(\alpha_{\bar{z}}\left(\partial_{r}\;-\;\frac{i}{r}\partial_{\theta }\;+\;\frac{\sqrt{\ell (\ell \;+\;1)}\;+\;\phi }{r}\;+\;i\frac{kr}{1\;-\;r^{2}}\right)\right),$$

for $k\;=\;BR^{2}/4$. With the approximation that r<<1 or small orbits the product gives the Klein-Gordon equation

$$\partial_{t}^{2}\psi \;=\;R^{-2}\left(\partial_{r}^{2}\;+\;\frac{\ell \left(\ell \;+\;1\right)\;+\;\phi^{2}}{r^{2}}\;+\;k^{2}r^{2}\;+\;\left(\ell \left(\ell \;+\;1\right)\;+\;\phi^{2}\right)k\right)\psi .$$

For $\ell \left(\ell \;+\;1\right)\;+\;\phi^{2}\;=\;0$ this gives the Weber equation with parabolic cylinder functions for solutions. The last term $(\ell \left(\ell \;+\;1\right)\;+\;\phi^{2})k$ can be absorbed into the constant phase $\psi \left(r,t\right)\;=\;\psi \left(r\right)e^{-it\sqrt{E^{2}\;+\;\ell \left(\ell \;+\;1\right)\;+\;\phi^{2}}}$. This dynamics for a particle in a Poincare disk is used to model the same dynamics for a particle in a region bounded by the event horizons of a black hole. With AdS black hole correspondence the field content of the AdS boundary is the same as the horizon of a black hole. An elementary way to accomplish this is to map the Poincare disk into a strip. The boundaries of the strip then play the role of the event horizons. The fields of interest between the horizons are assumed to have orbits or dynamics not close to the horizons. The map is z=tanh(ξ). The Klein-Gordon equation is then

(10) $$\partial_{t}^{2}\psi \;=\;R^{-2}\left(\left(1\;+\;2\xi^{2}\right)\partial_{\xi }\partial_{\bar{\xi }}\;+\;\frac{\ell \left(\ell \;+\;1\right)\;+\;\phi^{2}}{{\left|\xi \right|}^{2}}\;-\;k{\left|\xi \right|}^{2}\right)\psi ,$$

where the $\xi^{2}$ is set to zero under this approximation. The Klein-Gordon equation is identical to the above.

The solution to this differential equation for $\Phi \;=\;\ell \left(\ell \;+\;1\right)\;+\;\phi^{2}$ is

$$\psi \;=\;{\left(2\xi \right)}^{\frac{1}{4}\left(\sqrt{1\;-\;4\Phi }\;+\;1\right)}e^{-\frac{1}{2}k\xi^{2}}\times$$

$$\left[c_{1}U\left(\frac{1}{4}\left(\frac{E^{2}R^{2}}{k}\;+\;\sqrt{1\;-\;4\Phi }\;+\;1\right),\;\frac{1}{2}\left(\sqrt{1\;-\;4\Phi }\;+\;1\right),\;k\xi^{2}\right)\;+\;c_{2}L_{\frac{E^{2}R^{2}}{k}+\sqrt{1-4\Phi }}^{\frac{1}{2}\sqrt{1-4\Phi }}\left(k\xi^{2}\right)\right].$$

**Figure 1:**
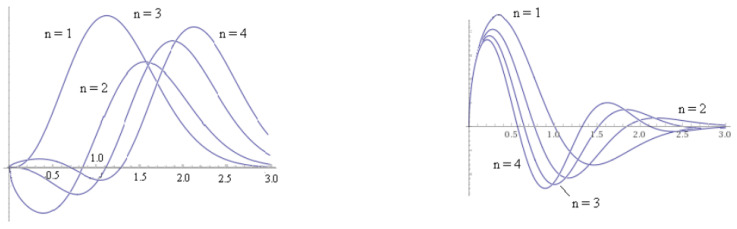
These are the wave function components contributed by the parabolic cylinder functions, or Hermite polynomials and the Laguerre polymomials. These depend on $x^{2}$ = k$\xi^{2}$ so the wave function is radial. These are not nomalized. (**left**) Solution of the form $x^{1/4}e^{-x^{2}}H_{n}\left(x^{2}\right)$ given by parabolic cylinder function for n = 1, 2, 3,...4 represented as a Hermite polynomial; (**right**) Laguerre wave function $x^{1/4}e^{-x^{2}}L_{n}^{0}\left(x^{2}\right)$ for hydrogen atomic-like states for n = 1,2,3,4.

The first of these is the confluent hypergeometric function of the second kind. For Φ=0 this reduces to the parabolic cylinder function. The second term is the associated Laguerre polynomial. The wave determined by the parabolic cylinder function and the radial hydrogen-like function have eigenmodes of the form in the diagram above. The parabolic cylinder function $D_{n}\;=\;2^{n/2}e^{-x^{2}/4}H_{n}\left(x/\sqrt{2}\right)$ with integer *n* gives the Hermite polynomial. The recursion formula then gives the modes for the quantum harmonic oscillator. The generalized Laguerre polynomial $L_{n-\ell -1}^{2\ell +1}\left(r\right)$ of degree n−ℓ−1 gives the radial solutions to the hydrogen atom. The associated Laguerre polynomial with general non-integer indices has degree associated with angular momentum and the magnetic fields. This means a part of this function is similar to the quantum harmonic oscillator and the hydrogen atom. The two parts in a general solution have amplitudes $c_{1}$ and $c_{2}$ and quantum states in between the close horizons of coalescing black holes are then in some superposition of these types of quantum states (See Figure 1).

The Hamiltonian

$$H\;=\;\frac{1}{2}{\left|\pi \right|}^{2}\;-\;\frac{g}{r^{2}},\;\pi \;=\;-i\partial_{r},$$

which contains the monopole field, describes the motion of a gauge particle in the hyperbolic space. In addition, there is a contribution from the constant magnetic field $U\;=\;-kr^{2}/2$. Now convert this theory to a scalar field theory with r→ϕ and $\pi \;=\;-i\partial_{r}\phi$. Finally introduce the dilaton operator *D* and the scalar theory consists of the operators

$$H_{0}\;=\;\frac{1}{2}{\left|\pi \right|}^{2}\;-\;\frac{g}{\phi^{2}},\;U\;=\;-k\frac{\phi^{2}}{2},\;D\;=\;\frac{1}{4}\left(\phi \pi \;+\;\pi \phi \right),$$

where $H_{0}\;+\;U$ is the field theoretic form of the potential in Equation (9). These potentials then lead to the algebra

$$\left[H_{0},\;U\right]\;=\;-2iD.\;\left[H_{0},\;D\right]\;=\;-iH_{0},\;\left[U,\;D\right]\;=\;iM.$$

This may be written in a more compact form with $L_{0}\;=\;2^{-1/2}\left(H_{0}\;+\;U\right)$, which is the total Hamiltonian, and $L_{\pm }\;=\;2^{-1/2}\left(U\;-\;H_{0}\;\pm \;iD\right)$. This leaves the SL(2,R) algebra

(11) $$\left[L_{0},\;L_{\pm }\right]\;=\;\pm iL_{0},\;\left[L_{+},\;L_{-}\right]\;=\;L_{0}.$$

This is the standard algebra ∼su(2). Given the presence of the dilaton operator this indicates conformal structure. The space and time scale as (t,x)→λ(t,x) and the field transforms as $\phi \;\to \;\lambda^{\Delta }\phi$. The measure of the integral $d^{4}x\sqrt{g}$ is invariant, where $\lambda \;=\;\partial x^{\prime }/\partial x$ gives the Jacobian $J\;=\;det|\frac{\partial x^{\prime }}{\partial x}|$ that cancels the $\sqrt{g}$ and the measure is independent of scale. In doing this we are anticipating this theory in four dimensions. We then simply have the scaling $\phi \;\to \;\lambda^{-1}\phi$ and π→π. For the potential term $-g/2\phi^{2}$ invariance of the action requires $g\;\to \;\lambda^{-2}g$ and for $U\;=\;-k\frac{\phi^{2}}{2}$ clearly $k\;\to \;\lambda^{2}k$. This means we can consider this theory for 2 space plus 1 time and its gauge-like group SL(2,R) as one part of an $SL\left(2,\;C\right)\;∼\;SL{\left(2,\;R\right)}^{2}$. The differential equation number 10 is a modified form of the Weber equation $\psi_{xx}\;-\;\left(\frac{1}{4}x^{2}\;+\;c\right)\psi \;=\;=0$ The solution in Abramowit and Stegun are parabolic cylinder functions $D_{-a-1/2}\left(x\right)$, written according to hypergeometric functions. The $\xi^{-1}$ part of the differential equation contributes the Laguerre polynomial solution. If we let $\xi \;=\;e^{x/2}$ and expand to quadratic powers we then have the potential in the variable x.

$$V\left(x\right)\;=\;-\left(g\;+\;k\right)\;+\;\frac{1}{2}\left(k\;-\;g\right)\left(x^{2}\;+\;x^{4}\right),$$

for *g* and *k* the constants in $H_{0}$ and *U*. The Schrodinger equation for this potential with a stationary phase in time has the parabolic cylinder function solution

$$\psi \left(x\right)\;=\;c_{1}D_{\frac{\beta^{2}-4\left(\alpha +2\sqrt{2}\alpha^{3/2}\right)}{16\sqrt{2}\alpha^{3/2}}}\left(\frac{\beta (1+4x)}{\sqrt{2}{\left(2\alpha \right)}^{3/4}}\right)\;+\;c_{2}D_{\frac{-\beta^{2}-4\left(\alpha -2\sqrt{2}\alpha^{3/2}\right)}{16\sqrt{2}\alpha^{3/2}}}\left(\frac{i\beta (1+4x)}{\sqrt{2}{\left(2\alpha \right)}^{3/4}}\right),$$

where α=g+k and β=k−g. The parabolic cylinder function describes a theory with criticality, which in this case has with a Ginsburg-Landau potential. The field theory form also has parabolic cylinder function solutions. The field theory with the field expanded as $\phi \;=\;e^{\chi }$ is expanded around unity so ϕ≃$1\;+\;\chi \;+\;\frac{1}{2}\chi^{2}$. A constant *C* such that χ→Cχ is unitless is assumed or implied to exist. The Lagrangian for this theory is

$$L\;=\;\frac{1}{2}\partial_{\mu }\chi \partial^{\mu }\chi \;+\;\alpha \;+\;\frac{1}{2}\mu^{2}\chi^{2}\;+\;2\beta \mu \chi .$$

The constant μ, standing for mass and absorbing α, is written for dimensional purposes. We then consider the path integral $Z\;=\;D\left[\chi \right]e^{-iS-i\chi J}$. Consider the functional differentials acting on the path integral

$$\left(\left(p^{2}\;+\;m^{2}\right)\frac{\delta }{\delta J}\;-\;2i\beta \right)Z\;=\;-i\langle \frac{\delta S}{\delta \chi }\rangle ,$$

where $\partial_{\mu }\chi \;=\;p_{\mu }\chi$. The Dyson-Schwinger theorem tells us that $\langle \frac{\delta S}{\delta \chi }\rangle \;=\;\langle J\rangle$ mean we have a polynomial expression $\langle \frac{1}{2}\left(p^{2}\;+\;m^{2}\right)\chi$−iβ−J〉=0, where we can trivially let J−iβ→J. This does not lead to parabolic cylinder functions. There has been a disconnect between the ordinary quantum mechanical theory and the QFT. We may however, continue the expansion to quartic terms. This will also mean there is a cubic term, we may impose that only the real functional variation terms contribute and so only even power of the field define the Lagrangian

$$L\;\to \;\frac{1}{2}\partial_{\mu }\chi \partial^{\mu }\chi \;+\;\alpha \;+\;\frac{1}{2}\mu^{2}\chi^{2}\;+\;\frac{1}{4}\lambda \chi^{4},$$

where $\frac{2}{3}\alpha \;\to \;\frac{1}{4}\lambda$. The functional derivatives are then

$$\left(\left(p^{2}\;+\;m^{2}\right)\frac{\delta }{\delta J}\;+\;\lambda \frac{\delta^{3}}{\delta J^{3}}\right)Z\;=\;-i\langle \frac{\delta S}{\delta \chi }\rangle ,$$

This cubic form has three parabolic cylinder solutions. We may think of this as $ap\;+\;bp^{3}\;=\;J$ and is a cubic equation for the source *J* that is annulled at three points. The correspond to distinct solutions with distinct paths. These three solutions correspond to three contours and define three distinct vacua. The overall action is a quartic function, which will have three distinct vacua, where one of these is the low energy physical vacua. It is worth noting this transformation of the problem has converted it into a system similar to the Higgs field. This system with both harmonic oscillator and a Coulomb potentials is conformal and it maps into a system with parabolic cylinder functions solutions. In effect there is a transformation harmonicoscillator states↔hydrogen−likestates. The three solutions would correspond to the continuance of conformal symmetry, but where the low energy vacuum for one of these may not appear to be conformally invariant. This scale transformation above is easily seen to be the conformal transformation with λ=Ω. The scalar tensor theory of gravity for coupling constant κ=16πG

(12) $$S\left[g,\;\phi \right]\;=\;\int d^{4}x\sqrt{g}\left(\frac{1}{\kappa }R\;+\;\frac{1}{2}\partial_{\mu }\phi \partial^{\mu }\phi \;+\;V\left(\phi \right)\right).$$

This then has the conformal transformations

$$g_{\mu \nu }^{\prime }\;=\;\Omega^{2}g_{\mu \nu },\;\phi^{\prime }\;=\;\Omega^{-1}\phi ,\;\Omega^{2}\;=\;1\;+\;\kappa \phi^{2}.$$

with the transformed action

(13) $$S\left[g^{\prime },\;\phi^{\prime }\right]\;=\;\int d^{4}x\sqrt{g^{\prime }}\left(\frac{1}{\kappa }R^{\prime }\;+\;\frac{1}{2}g^{\prime \mu \nu }\partial_{\mu }\phi^{\prime }\partial_{\nu }\phi^{\prime }\;+\;V\left(\phi^{\prime }\right)\;+\;\frac{1}{12}R\phi^{\prime 2}\right).$$

There is then a hidden SO(3,1)≃SL(2,C) symmetry. Given an internal index on the scalar field $\phi^{i}$ there is a linear SO(n) transformation $\delta \phi^{i}\;=$$C^{ijk}\phi_{j}\delta \tau_{k}$ for $\tau_{k}$ a parameter. There is also a nonlinear transformation from Equation (12) as $\delta \phi^{i}\;=\;{\left(1\;+\;\kappa \phi^{2}\right)}^{1/2}\kappa \delta \chi^{i}$ for $\chi^{i}$ a parameterization. In the primed coordinates the scalar field and metric transform as

$$\delta \phi^{i}\;=\;\delta \tau^{i}\;-\;\kappa \phi^{\prime i}\phi^{j}\delta \chi^{j}$$

(14) $$\delta g_{\mu \nu }\;=\;\frac{2g_{\mu \nu }^{\prime }\kappa \phi^{\prime i}\delta \chi^{i}}{1\;-\;\kappa \phi^{\prime 2}}.$$

The gauge-like dynamics have been buried into the scalar field. With this semi-classical model the scalar field adds some renormalizability. Further this model is conformal. The conformal transformation mixes the scalar field, which is by itself renormalizable, with the spacetime metric. Quantum gravitation is however difficult to renormalize. Yet we see the linear group theoretic transformation of the scalar field in SO(n) is nonlinear in SO(n,1). Conformal symmetry is manifested in sourceless spacetime, or spatial regions without matter or fields. The two dimensional spatial surface in $AdS_{3}$ is the Poincare disk that with complexified coordinates has metric with SL(2,R) algebraic structure. This may of course be easily extended into SL(2,C) as SL(2,R)×SL(2,R). In this conformal setting quantum states share features similar to the emission of photons by a harmonic oscillator or an atom. The orbits of these paths are contained in regions bounded by hyperbolic surfaces, or arcs for the two dimensional Poincare disk. The entropy associated with these arcs is a measure of the area contained within these curves. This is in a nutshell the Mirzakhani result on entropy for hyperbolic curves. This development is meant to illustrate how radiation from black holes is produced by quantum mechanical means not that different from bosons produced by a harmonic oscillator or atom. Hawking radiation in principle is detected with a wavelength not different from the size of the black hole. The wavelength approximately equal to the Schwarzschild radius has energy E=hν corresponding to a unit mass emitted. The mass of the black hole is *n* of these units and it is easy to find $m_{p}\;=$$\sqrt{\hbar c/G}$. These modes emitted are Planck units of mass-energy that reach $I^{\infty }$. In the case of gravitons, these carry gravitational memory. For the coalescence of black holes gravitational waves are ultimately gravitons. For Hawking radiation there is the metric back reaction, which in a quantum mechanical setting is an adjustment of the black hole with the emission of gravitons. The emission of Hawking radiation might then be compared to a black hole quantum emitting a Planck unit of black hole that then decays into bosons. The quantum induced change in the metric is a mechanism for producing gravitons. In the coalescence of black holes the quantum hair on the stretched horizons sets up a type of Casimir effect with the vacuum that generates quanta. In general these are gravitons. We might see this as not that different from a scattering experiment with two Planck mass black holes. These will coalesce, form a larger black hole, produce gravitons, and then quantum states excited by this process will decay. The production of gravitons by this mechanism is affiliated with normal modes in the production of gravitons, which in principle is not different from the production of photons and other particles by other quantum mechanical processes. I fact quantum mechanical processes underlying black hole coalescence might well be compared to nuclear fusion. The 2 LIGOs, plus now the VIRGO detector, are recording and triangulating the positions of distant black hole collisions almost weekly. This information may contain quantum mechanical information associated with quantum gravitation. This information is argued below to contain BMS symmetries or information. This will be most easily detected with a space-based system such as eLISA, where the shift in metric positions of test masses is most readily detectable. However, preliminary data with the gross displacement of the LIGO mass may give preliminary information as well.

## 5. Discussion

The coalescence of two black holes is a form of scattering. We may think of black holes as an excited state of the quantum gravity field and a sort of elementary particle. The scattering of two black holes results in a larger black hole plus gravitational radiation. This black hole will then emit Hawking radiation. Thus, in general the formation of black holes, their coalescence and ultimate quantum evaporation is an intermediate processes in a general scattering theory.

Quantum hair is a set of quantum fields that build up quantum gravitation, in the manner of gauge-gravity duality and BMS symmetry. This is holography, with the fields on the horizons of two BHs that determine the graviton/GW content of the BH coalescence. A detailed analysis of this may reveal BMS charges that reach $I^{+}$ are entangled with Hawking radiation by a form of entanglement swap. In this way Hawking radiation may not be entangled with the black hole and thus not with previously emitted Hawking radiation. This will be addressed later, but a preliminary to this idea is seen in Reference [23], for disentanglement between Hawking radiation and a black hole. The authors are working on current calculations where this is an entanglement swap with gravitons. The black hole production of gravitons in general is then a manifestation of quantum hair entanglement. It is illustrative for physical understanding to consider a linearized form of gravitational memory. Gravitational memory from a physical perspective is the change in the spatial metric of a surface according to Reference [3]

$$\Delta h_{+.\times }\;=\;\lim_{t\to \infty }h_{+,\times }\left(t\right)\;-\;\lim_{t\to -\infty }h_{+,\times }\left(t\right).$$

Here, + and *x* refer to the two polarization directions of the GW. See Reference [24] for more on this. Quantum hair on two black holes just before coalescence are highly excited and contribute to spacetime curvature, or in a full context of quantum gravitation the generation of gravitons. As yet there is no complete theory of quantum gravity, but it is reasonable to think of gravitational radiation as a classical wave built from many gravitons. Gravitons have two polarizations and a state $|\Psi_{+,\times }\rangle$ the density matrix $\rho_{+,\times }\;=$$|\Psi_{+,\times }\rangle \langle \Psi_{+,\times }|$ then defines entropy $S\;=\;\rho_{+,\times }log\left(\rho_{+,\times }\right)$ that with this near horizon condition of AdS with a black hole is a form of Mirzakhani entropy measure in hyperbolic space. The gravitons emitted are generated by quantum hair on the colliding black holes. These will contribute to gravitational waves, and in general with BMS translations that bear quantum information from quantum hair.

This theory connects to fundamental research, The entanglement entropy of $CFT_{2}$ entropy with $AdS_{3}$ lattice spacing *a* is

$$S\;≃\;\frac{R}{4G}ln\left(|\gamma |\right)\;=\;\frac{R}{4G}ln\left[\frac{\ell }{L}\;+\;e^{2\rho_{c}}sin\left(\frac{\pi \ell }{L}\right)\right].$$

where the small lattice cut off avoids the singular condition for ℓ=0 or *L* for $\rho_{c}\;=\;0$. For the metric in the form $ds^{2}\;=\;{\left(R/r\right)}^{2}\left(-dt^{2}\;+\;dr^{2}\;+\;dz^{2}\right)$ the geodesic line determines the entropy as the Ryu-Takayanagi (RT) result [25]

$$S\;=\;\frac{R}{2G}\int_{2\ell /L}^{\pi /2}\frac{ds}{sin\;s}\;=\;-\frac{R}{2G}ln\left[cot\left(s\right)\;+\;csc\left(s\right)\right]{|}_{2\ell /L}^{\pi /2}$$

$$≃\;\frac{R}{2G}ln\left(\frac{\ell }{L}\right),$$

which is the small *ℓ* limit of the above entropy. The RT result specifies entropy, which is connected to action $S_{a}\;↔\;S_{e}$ [26]. Complexity, a form of Kolmogoroff entropy [27], is $S_{a}/\pi \hbar$ which can also assume the form of the entropy of a system S∼klog(dimH) for H the Hilbert space and the dimension over the number of states occupied in the Hilbert space. There is also complexity as the volume of the Einstein-Rosen bridge [28] $vol/GR_{ads}$ or equivalently the RT area $∼\;vol/R_{AdS}$. There is an equivalency between entropy or complexity according to the geodesic paths in hyperbolic $H^{2}$ by geometric means [21]. This should generalize to $H^{3}$$\subset \;AdS_{4}$. The generation of gravitational waves should have an underlying quantum mechanical basis. It is sometimes argued that spacetime physics may not be at all quantum mechanical. This is probably a good approximation for energy sufficient orders of magnitude lower than the Planck scale. However, if we have a scalar field that define the metric $g^{\prime }\;=\;g^{\prime }\left(g,\;\phi \right)$ with action S[g,ϕ] then a quantum field ϕ and a purely classical *g* means the transformation of *g* by this field has no quantum physics. In particular for a conformal theory $\Omega \;=\;1\;+\;\kappa \phi^{a}\phi^{a}$, here *a* an internal index, the conformal transformation $g_{\mu \nu }^{\prime }\;=\;\Omega^{2}g_{\mu \nu }$ has no quantum content. This is an apparent inconsistency. For the inflationary universe the line element

$$ds^{\prime 2}\;=\;g_{\mu \nu }^{\prime }dx^{\mu }dx^{\nu }\;=\;\Omega^{2}\left(du^{2}\;-\;d\Sigma^{\left(3\right)}\right),$$

with $dt/du\;=\;\Omega^{2}$ gives an FLRW or de Sitter-like line element that expands space with $\Omega^{2}\;=\;e^{t\sqrt{\Lambda /3}}$. The current slow accelerated universe we observe is approximately of this nature. The inflation scalars are then fields that stretch space as a time dependent conformal transformation and are quantum mechanical. The generation of gravitational waves is ultimately the generation of gravitons. Signatures of these quantum effects in black hole coalescence will entail the measurement of quantum information. Gravitons carry BMS charges and these may be detected with a gravitational wave interferometer capable of measuring the net displacement of a test mass. The black hole hair on the stretched horizon is excited by the merger and these results in the generation of gravitons. The Weyl Hamiltonians in Equation (9) depend on the curvature as $\;\propto \;\sqrt{R}$. For the curvature extreme during the merging of black holes this means many modes are excited. The two black holes are pumped with energy by the collision, this generates or excites more modes on the horizons, where this results in a black hole with a net larger horizon area. This results in a metric response, or equivalently the generation of gravitons. Quantum normal modes are given by independent eigen-states, such as with quantum harmonic oscillator states. The harmonic oscillator states are well known to be given by the Hermite polynomials, which are a special case of parabolic cylinder functions. Rydberg states are also a form of normal modes. The quantum states for the hyperbolic geometry of black hole mergers are a generalization of these forms of states. The excitation of quantum hair in such a merger and the production of gravitons is a converse situation for the emission of Hawking radiation. In both cases there is a dynamical response of the metric, which is associated with gravitons. Currently a “by hand” correction called back reaction is used in models. A more explicit discussion on the production of gravitons is beyond the scope here. However, the parabolic cylinder functions and the Laguerre functions clearly play a role in quantum production of gravitons in BH coalescence. This means quantum gravitation should have signatures of much the same physics as atomic physics or the role of electrons and phonons in solids. The major import of this expository is to propose quantum gravitational signatures in the coalescence of black holes. This would point to quantum hair and the generation of gravitons. This would be a clear signature of quantum gravitation. While there is plenty of further development needed to compute more firm predictions, the generic result is that gravitational waves from colliding black holes have some quantum gravitational signatures. These signatures are to be found in gravitational memory. Further, this long-term adjustment of spacetime metric deviates form a purely classical expected result. With further advances in gravitational wave interferometry, in particular with the future eLISA space mission, it should be possible to detect elements of gravitons and quantum gravitation.
